# Neonatal Regulatory T Cells Mediate Fibrosis and Contribute to Cardiac Repair

**DOI:** 10.3390/cells15020204

**Published:** 2026-01-22

**Authors:** Tabito Kino, Sadia Mohsin, Yumi Chiba, Michiko Sugiyama, Tomoaki Ishigami

**Affiliations:** 1 Department of Cardiology, Yokohama City University, Yokohama 236-0004, Japan; 2 Cardiovascular Research Center, Lewis Katz School of Medicine, Temple University, Philadelphia, PA 19140, USA; 3 Advanced Adult Nursing, Department of Nursing, Graduate School of Medicine, Yokohama City University, Yokohama 236-0004, Japan

**Keywords:** neonatal heart, myocardial infarction, cardiac repair, CD4+Foxp3+ T (T-reg) cells, Rcn3, PI3K/Akt pathway, fibrosis, immune homeostasis

## Abstract

**Highlights:**

**What are the main findings?**

- Neonatal hearts display a distinct post-infarction immune profile characterized by the accumulation of CD4+Foxp3+ T (T-reg) cells with reparative transcriptional programs.
- Reticulocalbin 3 (Rcn3) is selectively upregulated in neonatal T-reg cells and is required for functional recovery and suppression of fibrosis after myocardial infarction.

**What are the implications of the main findings?**

- Neonatal T-reg cells actively contribute to cardiac repair by modulating endoplasmic reticulum stress responses and paracrine anti-fibrotic signaling.
- Targeting T-cell-specific pathways such as Rcn3 may represent a novel immunomodulatory strategy to enhance myocardial repair in the adult heart.

**Abstract:**

The neonatal heart possesses a unique capacity for reparative healing after myocardial injury, unlike the adult heart. While immune cells, particularly T cells, regulate post-infarction inflammation, their role in age-dependent cardiac repair remains unclear. This study aimed to characterize the temporal activation of T cell subsets and their contribution to immune homeostasis and myocardial repair. Myocardial infarction was induced in mice of different ages, and T cell subsets (CD4+ T cells, CD8+ T cells, and CD4+Foxp3+ T [T-reg] cells) were analyzed using flow cytometry and RNA sequencing. Neonatal hearts exhibited CD4+ T cells, CD8+ T cells, and T-reg cells that gradually increased until seven days post-injury. Transcriptome analysis identified Rcn3 as a neonatal-specific, injury-responsive gene in T-reg cells, with minimal induction in adult and aged hearts, promoting a reparative microenvironment and exerting anti-fibrotic effects via the PI3K/Akt pathway. Under endoplasmic reticulum stress, Rcn3 activated unfolded protein response genes, and Rcn3-conditioned media reduced fibrosis-associated gene expression in adult cardiac fibroblasts. In a conditional knockout mouse model (Lck-cre; Rcn3^fl/fl^), Rcn3 deletion in T cells led to impaired cardiac function recovery and increased fibrosis post-injury. These findings suggest that neonatal T-reg cells play a crucial role in cardiac repair, with Rcn3 as a potential therapeutic target for enhancing immune-mediated cardiac repair and limiting pathological remodeling in the adult heart.

## 1. Introduction

Ischemic heart disease remains the leading cause of death worldwide [1]. Myocardial infarction (MI) initiates a cascade of pathological processes, including inflammation, adverse ventricular remodeling, and fibrotic scar formation, which frequently progresses to cardiac dysfunction and heart failure. Identifying mechanisms that enhance myocardial repair after ischemic injury therefore remains a major objective in cardiovascular research.

Accumulating evidence indicates that the mammalian heart possesses a transient regenerative capacity during early postnatal life. In mice, robust myocardial regeneration occurs until approximately postnatal day 7 (P7) following injury induced by apical resection or coronary ligation [2,3]. Comparable regenerative responses have been reported in neonatal pigs and humans, characterized by robust angiogenesis and minimal fibrosis [4,5,6,7]. This reparative capacity is rapidly lost after P7 in rodents, after which myocardial injury results in permanent scarring and sustained functional impairment [2,3].

Fate-mapping studies have demonstrated that neonatal cardiac regeneration primarily results from the proliferation of pre-existing cardiomyocytes rather than from progenitor or stem cell populations [8,9]. While cardiomyocyte-intrinsic mechanisms have been extensively studied, growing evidence suggests that immune responses are also critical determinants of post-infarction outcomes. The immune system orchestrates the initiation and resolution of inflammation after MI and plays a central role in shaping scar formation and left ventricular remodeling [10,11,12]. Among adaptive immune cells, T lymphocytes have emerged as potential regulators of cardiac repair [13]; however, how age-dependent differences in T cell composition and function influence regenerative versus fibrotic healing remains poorly understood. Using unbiased transcriptomic profiling of cardiac T cell subsets after myocardial infarction, we identified reticulocalbin 3 (Rcn3) as one of the most selectively and robustly upregulated genes in neonatal CD4+Foxp3+ T (T-reg) cells. Notably, this injury-induced upregulation of Rcn3 was prominent in neonatal hearts but minimal or absent in adult and aged hearts, suggesting an age-dependent divergence in Rcn3 regulation.

In particular, interactions between distinct T cell subsets and cardiac-resident cells, such as fibroblasts and cardiomyocytes, may determine whether myocardial injury resolves through repair or progresses toward fibrosis. We hypothesized that differences in the prevalence, activation, and downstream signaling of T cell subsets between neonatal and aged hearts critically influence post-infarction repair. Based on these observations, and on our unbiased transcriptomic identification of Rcn3 as a neonatal-specific, injury-responsive gene in T-reg cells, this study aimed (1) to characterize the temporal dynamics of T cell subsets following MI in neonatal versus aged mice, and (2) to determine how T-cell-mediated paracrine signaling regulates myocardial repair in an age-dependent manner.

## 2. Materials and Methods

### 2.1. Animals

All animal procedures were approved by the Institutional Animal Care and Use Committee of the Lewis Katz School of Medicine at Temple University (protocol #5060, approved 21 March 2022) and conducted in accordance with AAALAC guidelines. Mice were housed under controlled environmental conditions (22–24 °C, 12 h light/dark cycle) with unrestricted access to food and water. C57BL/6J mice and B6.Cg-Tg(Lck-cre)548Jxm/J mice were obtained from The Jackson Laboratory (#000664 and #003802, Bar Harbor, ME, USA) [14]. Rcn3^fl/fl^ mice were provided by Dr. Kyu Sang Joeng (University of Pennsylvania, Philadelphia, PA, USA) [15]. Adult and aged experiments were performed using male mice only to minimize potential variability related to sex differences. Neonatal mice were used without sex discrimination, as sex cannot be reliably determined at postnatal day 1. The experimental unit was a single mouse. Because cardiac T cells are virtually absent under steady-state conditions, sham-operated groups were not included in the present study. This design was based on prior reports demonstrating minimal accumulation of T cells in sham-operated myocardium and was intended to focus specifically on injury-induced immune dynamics [16].

### 2.2. Myocardial Infarction Surgery

Permanent myocardial infarction (MI) was induced by ligation of the left anterior descending (LAD) coronary artery as previously described [17,18]. For neonatal MI, postnatal day 1 (P1) mice were anesthetized by hypothermia on an ice bed. Following skin incision, a lateral thoracotomy was performed at the fourth intercostal space by blunt dissection. The LAD was ligated using an 8-0 non-absorbable suture (#T06A08N14-13, AROSurgical, Newport Beach, CA, USA). Successful ischemia was confirmed by regional myocardial pallor. The chest wall was closed with an 8-0 suture, and the skin was sealed using tissue adhesive (#1469SB, 3M, St. Paul, MN, USA). For adult (8-week-old) and aged (1-year-old and 2-year-old) mice, anesthesia was induced with inhaled isoflurane (0.5–2%), and the mice were mechanically ventilated with an oxygen mixture. A left thoracotomy was performed through the fourth intercostal space, and the pectoralis muscles were transected to expose the thoracic cavity. After opening the pericardium, the LAD was ligated with a 7-0 nylon suture (#1647G, Ethicon, Somerville, NJ, USA). Ischemia was verified by pallor of the anterior left ventricular wall. The thoracotomy was closed in layers using 5-0 silk sutures (#K870H, Ethicon). Animals were monitored daily for general health and signs of distress. No unexpected adverse events occurred, and no predefined humane endpoints were reached during the study.

### 2.3. Transthoracic Echocardiography

Transthoracic echocardiography (TTE) was performed in accordance with established guidelines [19]. Mice were anesthetized with 1–3% isoflurane and positioned on a heated imaging platform to maintain body temperature. Cardiac imaging was conducted using a Vevo2100 ultrasound system equipped with an MS400 transducer (VisualSonics, Tronto, ON, Canada). B- and M-mode images were acquired and analyzed using VevoLab software version 3.2.5 (VisualSonics). Parameters assessed included interventricular septal thickness, anterior and posterior wall thickness, left ventricular internal diameter, end-diastolic and end-systolic volumes, stroke volume, fractional shortening, and ejection fraction (EF). TTE assessments were performed at baseline and at 1, 7, 14, and 21 days post-injury (dpi). An EF < 40% at 1 dpi was used to define successful infarction, and only these mice were included in subsequent analyses.

### 2.4. Immune Cell Isolation from Heart Tissue

For all analyses, hearts were extensively perfused to remove circulating blood cells, and immune cells were isolated from whole-heart tissue without regional dissection into infarct core, border zone, or remote myocardium. Hearts were harvested under anesthesia with 2.5–3% isoflurane and minced into small fragments. Tissue was digested in enzymatic buffer containing 20 mM HEPES, 450 U/mL Collagenase Type I (#LS004197, Worthington, Lakewood, NJ, USA), 125 U/mL Collagenase Type XI (#C7657, Sigma-Aldrich, St. Louis, MO, USA), 60 U/mL Deoxyribonuclease I (#LS002060, Worthington), and 60 U/mL Hyaluronidase (#LS005474, Worthington). Digestion was carried out at 37 °C for 40 min with continuous rotation. The resulting cell suspension was filtered through a 70 µm mesh and treated with red blood cell lysis buffer (155 mM NH_4_Cl, 10 mM KHCO_3_, 100 µM EDTA) for 10 min. Cells were washed and resuspended in sorting buffer consisting of 25 mM HEPES, 1 mM EDTA, and 4% fetal bovine serum in cation-free HBSS prior to flow cytometric staining and analysis.

### 2.5. T-Cell Population Characterization and Flow Cytometry

Flow cytometry was performed to characterize cardiac T-cell populations after MI. Single-cell suspensions isolated from heart tissue were stained with Live/Dead Fixable Green (#L23101, Thermo Fisher Scientific, Waltham, MA, USA), APC-eFluor780-conjugated anti-CD45 (30-F11; #47-0451-80, eBioscience, San Diego, CA, USA), PE-conjugated anti-CD3 (145-2C11; #100205, BioLegend, San Diego, CA, USA), PerCP-conjugated anti-CD4 (GK1.5; #100431, BioLegend), PE-Cy7-conjugated anti-CD8a (53-6.7; #25-0081-81, eBioscience), and eFluor450-conjugated anti-Foxp3 (FJK-16s; #48-5773-80, eBioscience). Cells were washed with phosphate-buffered saline, followed by live/dead staining for 30 min. Surface marker staining was performed for 20 min at 4 °C. For intracellular Foxp3 staining, cells were fixed and permeabilized using the True-Nuclear Fixation and Permeabilization Kit (#424401, BioLegend) according to the manufacturer’s instructions, followed by antibody incubation for 20 min. Flow cytometric acquisition and cell sorting of CD4+ T cells, CD8+ T cells, and CD4+Foxp3+ T-reg cells were performed using an LSRII Flow Cytometer and a FACSAria II (BD Biosciences, San Jose, CA, USA). Data were analyzed using FlowJo software version 10.8 (BD Biosciences).

### 2.6. RNA Sequencing and Transcriptomic Analysis

RNA was extracted from the same whole-heart preparations described above. RNA library preparation and sequencing were performed by Azenta US Inc. (Burlington, MA, USA). Ultra-low-input RNA sequencing libraries were generated using the SMART-Seq HT kit (#634455, Takara, Kusatsu, Shiga, Japan) for full-length cDNA synthesis and amplification. Sequencing libraries were prepared using the Nextera XT DNA Library Preparation Kit (#FC-131-1024, Illumina, San Diego, CA, USA). Briefly, amplified cDNA was fragmented and adapter-ligated using transposase-mediated tagmentation, followed by limited-cycle PCR for index incorporation. Library quality was assessed using an Agilent TapeStation and quantified using a Qubit 2.0 Fluorometer and quantitative PCR (KAPA Biosystems, Wilmington, MA, USA). Libraries were multiplexed and sequenced using a 2 × 150 bp paired-end configuration on an Illumina HiSeq 4000 platform (Illumina).

Raw base call files were converted to FASTQ format and demultiplexed using bcl2fastq version 2.17. Transcript quantification was performed against the mouse reference transcriptome (GRCm39) using Salmon version 1.2.0 [20]. Differential gene expression analysis was conducted using DESeq2 version 1.30.1 [21] in RStudio (version 2022.07.2+576; Posit, Boston, MA, USA). Genes with <0.5 counts per million were excluded. Differentially expressed genes were defined by an absolute fold change >2.0 and a false discovery rate <0.1.

Gene Ontology enrichment analysis, k-means clustering (four clusters, 12,000 genes), and bicluster analysis of the 1000 most variable genes were performed to identify biological processes associated with each T cell subset.

### 2.7. Adult Cardiac Fibroblast Isolation

Adult cardiac fibroblasts (ACFs) were used as a reductionist system to examine direct paracrine effects of T-cell-derived factors, rather than to model the neonatal post-MI microenvironment. Primary ACFs were isolated as previously described [22]. Briefly, minced heart tissue was enzymatically digested in HBSS containing 0.1% trypsin (#27250018, Gibco, Grand Island, NY, USA) and 100 IU/mL Collagenase II (#LS004174, Worthington). Cells were cultured at 37 °C in a humidified atmosphere with 5% CO_2_ in DMEM/F12 supplemented with 10% fetal bovine serum, 1% penicillin–streptomycin–glutamine (#10378-016, Gibco), and 100 µM 2-mercaptoethanol (#M3148, Sigma-Aldrich). ACFs at passage four were used for all experiments.

### 2.8. Lentiviral Transduction of Jurkat Cells

Jurkat cells (#TIB-152, ATCC, Manassas, VA, USA) were transduced with recombinant lentivirus encoding mRcn3 (pLV[Exp]-mCherry-CMV>mRcn3 [NM_026555.2]; Vector Builder, Chicago, IL, USA) or control vector (pLV[Exp]-CMV>mCherry; Vector Builder) at a multiplicity of infection of 40 in complete culture medium (#30-2001, ATCC) containing 0.1% polybrene (#TR-1003-G, EMD Millipore, Burlington, MA, USA). Transduction efficiency was assessed 72 h after infection, and mCherry-positive cells were sorted using an Influx cell sorter (BD Biosciences).

### 2.9. Rcn3 Treatment Under Endoplasmic Reticulum (ER) Stress Conditions

For ER stress induction, Jurkat cells were treated with thapsigargin (#T9033, Sigma-Aldrich) at 0.5 µM for 5 h. Recombinant human Rcn3 protein (#ab123203, Abcam, Cambridge, UK) was applied at concentrations of 1 µg/mL or 5 µg/mL where indicated. Conditioned media from Rcn3-transduced Jurkat cells under ER stress were transferred to ACF cultures and incubated for 24 h. To assess signaling pathway involvement, ACFs were pretreated for 1 h with PD98059 (#9900, Cell Signaling, Danvers, MA, USA) or wortmannin (#9951, Cell Signaling) at 10 µM prior to conditioned media exposure.

### 2.10. Quantitative Real-Time PCR

Total RNA was extracted using the RNeasy Kit (#74106, Qiagen, Hilden, Germany). Reverse transcription was performed using the High-Capacity cDNA Reverse Transcription Kit (#4368814, Applied Biosystems, Foster City, CA, USA). Quantitative PCR was conducted using a CFX96 Real-Time PCR System (Bio-Rad, Hercules, CA, USA). Relative gene expression was calculated using the ∆∆Ct method. Primer sequences are listed in Table S1.

### 2.11. Western Blot Analysis

Protein extraction and Western blotting were performed as previously described [23]. Protein concentrations were determined using a bicinchoninic acid assay (#23227, Thermo Fisher Scientific). Proteins were separated on Mini-PROTEAN TGX gels (#4561096, Bio-Rad) and transferred to nitrocellulose membranes (#1620115, Bio-Rad). Primary antibodies against Rcn3 (1:1000; #PA5-98793, Invitrogen, Carlsbad, CA, USA), Col1a1 (1:1000; #72026, Cell Signaling), phospho-Akt (Ser473) (1:2000; #4060, Cell Signaling), Akt (1:1000; #9272, Cell Signaling), β-actin (1:1000; #sc-517582, Santa Cruz Biotechnology, Dallas, TX, USA), and GAPDH (1:1000; #ab181602, Abcam and 1:200; #MAB374, Sigma-Aldrich) were incubated overnight at 4 °C. IRDye 800CW (1:1000; #926-32213, LI-COR Biosciences, Lincoln, NE, USA) and 680RD (1:1000; #926-68072, LI-COR Biosciences) secondary antibodies were used, and signals were detected using the Odyssey CLx imaging system (LI-COR Biosciences). Densitometric analysis was performed using Image Studio software (version 4.0).

### 2.12. Histological Analyses

Whole hearts were harvested, fixed in 4% paraformaldehyde at 4 °C overnight, and embedded in paraffin. Serial sections (5 μm thickness) were prepared for histological evaluation. Fibrotic remodeling was assessed using Masson’s trichrome staining performed with the Trichrome Stain (Masson) Kit (#HT15-1KT, Sigma-Aldrich) and Weigert’s Iron Hematoxylin Solution (#HT1079-1SET, Sigma-Aldrich), following the manufacturer’s protocols. Briefly, sections were incubated in Bouin’s solution (#HT10132, Sigma-Aldrich), stained with Weigert’s iron hematoxylin, Biebrich scarlet–acid fuchsin, phosphotungstic/phosphomolybdic acid, and aniline blue. Stained sections were imaged using an SMZ1000 light microscope (Nikon, Tokyo, Japan). Infarct size was quantified as the percentage of fibrotic scar length relative to the total left ventricular circumference, averaged from five sections per heart, using ImageJ software (version 1.53t; NIH, Bethesda, MD, USA) [24].

### 2.13. Statistical Analysis

Data are presented as mean ± standard deviation. Statistical analyses were performed using SPSS Statistics version 26 (IBM, Armonk, NY, USA). Comparisons between two groups were conducted using unpaired two-tailed Student’s *t*-tests. Comparisons among three or more groups were performed using one-way or two-way analysis of variance (ANOVA) with Tukey’s post hoc multiple-comparison tests where appropriate. Repeated measurements were analyzed using two-way repeated-measures ANOVA. A two-tailed *p* value < 0.05 was considered statistically significant. Data distribution and variance were assessed where appropriate; when assumptions for parametric tests were not met, non-parametric alternatives were applied.

Sample size was not determined by a formal a priori power calculation. This study was designed as an exploratory mechanistic investigation. For each experiment, the exact n (biological replicates; individual mice or independent cell preparations) is reported in the corresponding figure and/or Results. Sample sizes were chosen based on prior studies using similar neonatal myocardial infarction models and outcome measures, pilot data, and practical considerations, and were sufficient to detect biologically meaningful differences while minimizing animal use. Randomization was not performed, as experimental group allocation was determined by genotype and age. Potential confounders were minimized by standardized experimental procedures, and outcome assessment (e.g., echocardiographic and histological quantification) and data analysis were performed in a blinded manner.

## 3. Results

### 3.1. Age-Dependent Dynamics of Cardiac T Cells After Myocardial Infarction

**Figure 1:**
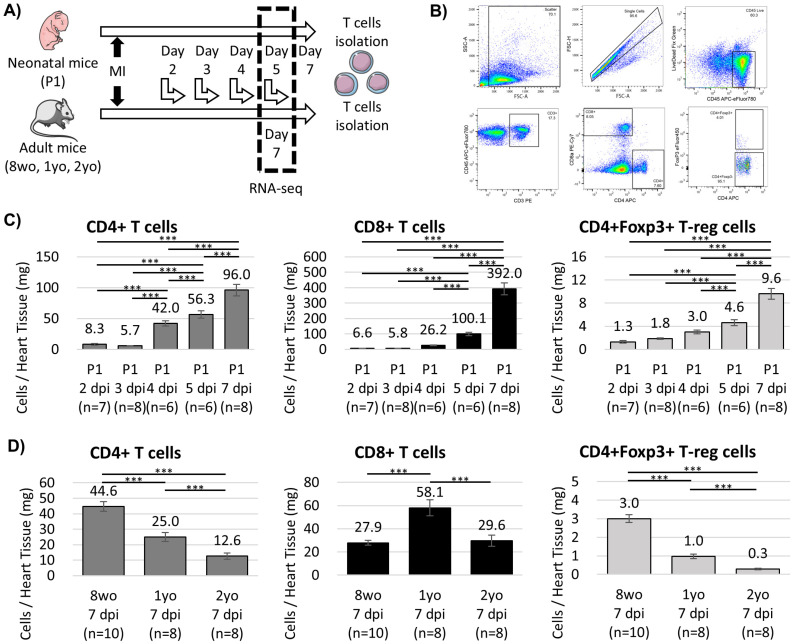
Age-dependent dynamics of cardiac T cells after myocardial infarction (MI). (**A**) Experimental overview. (**B**) Flow cytometric gating strategy. T-cell infiltration in (**C**) neonatal and (**D**) adult and aged hearts. Data represent mean ± SD and n indicates the number of individual mice per group. P1, postnatal day 1; 8wo, 8-week-old; 2yo, 2-year-old; dpi, days post-injury. *** *p* < 0.001.

We first examined the temporal accumulation of cardiac T cell subsets following MI in neonatal, adult, and aged mice (Figure 1A). Single-cell suspensions from injured hearts were analyzed by flow cytometry, and CD45+CD3+ cells were further classified into CD4+ T cell, CD8+ T cell, and CD4+Foxp3+ T-reg cell populations (Figure 1B).

In neonatal hearts, all T cell subsets progressively increased after injury and reached maximal levels by seven days post-injury (dpi) (Figure 1C). Among these, CD4+ T cells peaked earlier at 4 dpi, whereas CD4+Foxp3+ T-reg cells remained less abundant and declined by 7 dpi (Figure S1A). CD8+ T cells continued to expand and became the predominant T cell population at later time points.

Given that T-cell infiltration peaks around 7 dpi in adult hearts [16], we compared T-cell abundance across age groups at this time point. The number and frequency of CD4+ T cells and CD4+Foxp3+ T-reg cells decreased progressively with age. In contrast, CD8+ T cells were most abundant in aged hearts, peaking at one year of age and remaining elevated at two years (Figure 1D and Figure S1B). These data indicate a marked age-dependent shift in cardiac T-cell composition following MI.

### 3.2. Transcriptomic Profiling Reveals Distinct T-Cell Programs in Neonatal Versus Aged Hearts

To investigate age-specific functional programs, CD4+ T cells, CD8+ T cells, and CD4+Foxp3+ T-reg cells were isolated from 27 neonatal hearts at 5 dpi, 8 adult hearts (8 weeks old) at 7 dpi, and 8 aged hearts (2 years old) at 7 dpi, and pooled within each age group to generate RNA-seq libraries for each T cell subset. Transcriptomic analyses revealed broader enrichment of Gene Ontology (GO) terms in neonatal T cells compared with aged counterparts (Figure S2).

**Figure 2:**
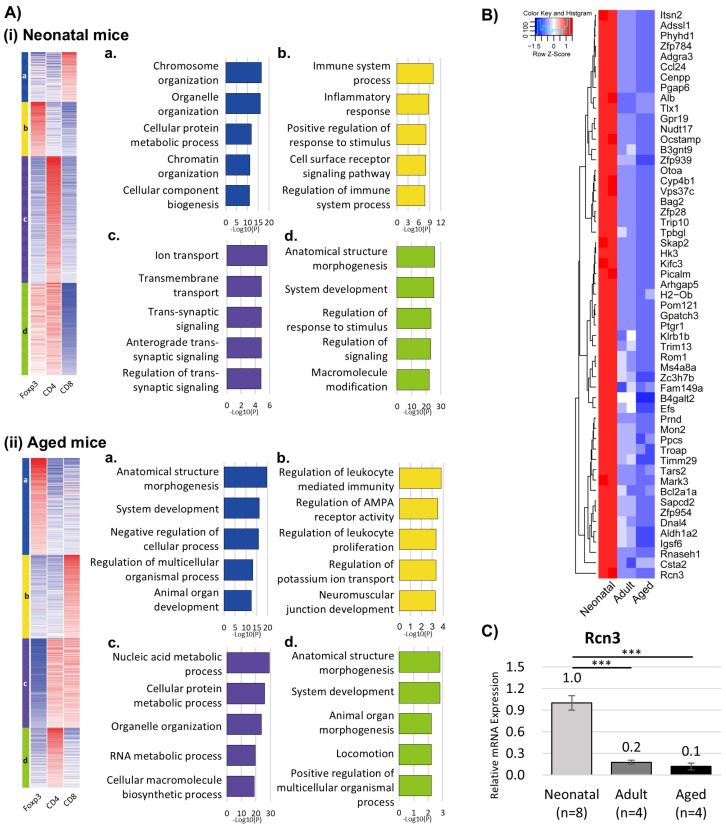
Transcriptome changes post-cardiac injury. (**A**) Cluster analysis of CD4+ T cells, CD8+ T cells, and CD4+Foxp3+ T-reg cells isolated from (**i**) neonatal mice and (**ii**) aged mice post-cardiac injury. (**B**) Heatmap of the upregulated genes in neonatal T-reg cells. (**C**) Rcn3 gene expression in whole-heart tissue from neonatal, adult, and aged mice. *** *p* < 0.001.

In neonatal hearts, common GO categories across T cell subsets included myeloid leukocyte migration and chemokine-mediated signaling pathways, whereas aged T cells were predominantly associated with adaptive immune responses and T cell-mediated immunity. K-means clustering further demonstrated subtype-specific functional signatures (Figure 2A). CD4+ T cells and CD4+Foxp3+ T-reg cells in neonatal hearts showed enrichment of GO terms related to anatomical structure morphogenesis and system development, whereas CD8+ T cells were primarily associated with ion transport and chromosome organization.

Comparative differential expression analysis revealed substantial upregulation of genes in neonatal T cells relative to aged cells, including 5294 genes (22.2%) in CD4+ T cells, 2903 genes (13.1%) in CD8+ T cells, and 5785 genes (25.3%) in CD4+Foxp3+ T-reg cells (Figure S3). Notably, genes associated with organelle organization and intracellular regulation were particularly enriched in neonatal CD4+Foxp3+ T-reg cells.

Among the genes selectively upregulated in neonatal CD4+Foxp3+ T-reg cells, Rcn3 emerged as a candidate factor associated with cardiac repair (Figure 2B). Rcn3 expression was markedly elevated in neonatal CD4+Foxp3+ T-reg cells during the acute phase after MI (Figure S4) and was also increased at the whole-heart level (Figure 2C).

### 3.3. Intracellular Rcn3 Enhances Endoplasmic Reticulum (ER) Stress Responses in T Cells

Given that Rcn3 is an ER-resident protein implicated in protein folding and trafficking [25,26], we examined its role under ER stress conditions. Thapsigargin-induced ER stress activated the unfolded protein response (UPR) in Jurkat T cells, as evidenced by increased activation of the spliced XBP1, ATF4, and ATF6 pathways (Figure S5A,B).

**Figure 3:**
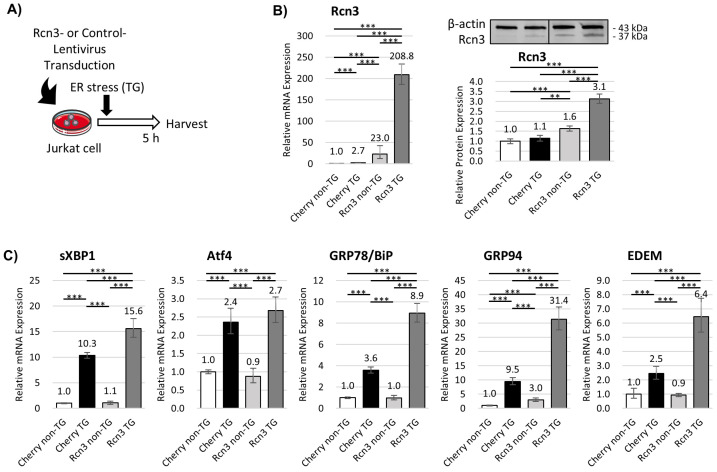
Endoplasmic reticulum (ER) stress response in Jurkat cells. (**A**) Experimental overview. (**B**) Rcn3 gene and protein expression under ER stress. (**C**) Expression of ER stress-responsible genes in control and Rcn3-transducted Jurkat cells. Data represent mean ± SD from n = 3 independent biological replicates. n indicates independent experiments performed on separate days. TG, thapsigargin. ** *p* < 0.01, and *** *p* < 0.001.

Extracellular treatment with recombinant Rcn3 did not significantly alter UPR target gene expression. In contrast, lentiviral overexpression of Rcn3 in Jurkat cells resulted in a marked increase in both Rcn3 mRNA and protein levels, particularly under ER stress conditions (Figure 3A,B and Figure S6). ER stress-responsive transcripts downstream of the IRE1 and ATF6 pathways were significantly upregulated in Rcn3-transduced cells compared with controls, whereas ATF4 expression was not further enhanced (Figure 3C). These findings suggest that intracellular Rcn3 potentiates UPR signaling in T cells during ER stress.

### 3.4. Rcn3 Suppresses Fibrotic Responses in Adult Cardiac Fibroblasts In Vitro

**Figure 4:**
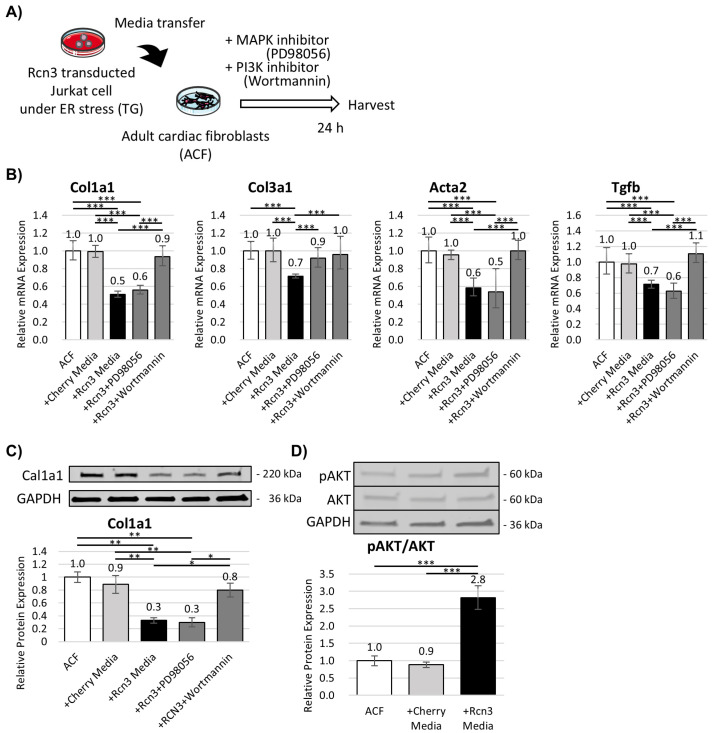
Effects of Rcn3-conditined media on adult cardiac fibroblasts (ACFs). (**A**) Experimental overview. (**B**) Expression of fibrosis-associated genes in ACFs and (**C**) Collagen I protein expressions in ACFs after treatment with control or Rcn3-conditioned media. (**D**) PI3K/Akt pathway activation in ACFs following treatment with Rcn3-conditioned media. Data represent mean ± SD from n = 3 independent biological replicates. n indicates independent experiments performed on separate days. TG, thapsigargin. * *p* < 0.05, ** *p* < 0.01, and *** *p* < 0.001.

Because Rcn3 has been reported to exert anti-fibrotic effects in human fibroblasts [27], we next assessed its impact on murine ACFs. Conditioned media from Rcn3-transduced Jurkat cells subjected to ER stress were applied to ACF cultures (Figure 4A).

After 24 h of treatment, expression of fibrosis-associated genes was significantly reduced in ACFs exposed to Rcn3-conditioned media (Figure 4B). Pharmacological inhibition revealed that this anti-fibrotic effect was abolished by the PI3K inhibitor wortmannin but not by the MAPK inhibitor PD98059. Consistently, collagen I protein expression was suppressed in ACFs treated with Rcn3-conditioned media, accompanied by increased Akt phosphorylation (Figure 4C,D). These results indicate that Rcn3-dependent paracrine signaling from T cells attenuates fibrotic responses in cardiac fibroblasts via the PI3K/Akt pathway.

### 3.5. T-Cell-Specific Deletion of Rcn3 Impairs Neonatal Cardiac Repair In Vivo

**Figure 5:**
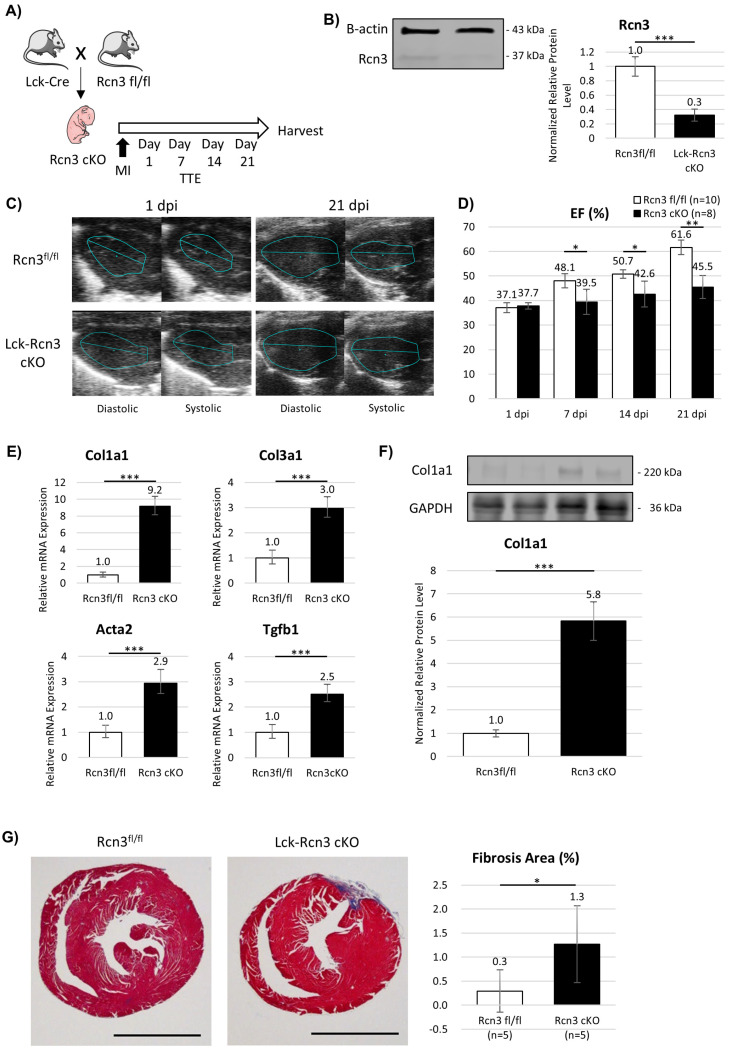
Phenotype of T cell-specific Rcn3 conditional knockout (cKO) mice. (**A**) Experimental overview. (**B**) Rcn3 protein expression in the spleen. (**C**) Representative images of the transthoracic echocardiogram (TTE) at 1 and 21 days post-injury (dpi). (**D**) TTE analyses of cardiac function, including left ventricular ejection fraction (EF). Fibrosis-associated (**E**) gene and (**F**) protein expressions. (**G**) Histological analyses using Masson’s Trichrome staining. The scale bar in each figure represents 2000 μm. Infarct size was quantified from five sections per heart and averaged per mouse. n indicates individual mice. MI, myocardial infarction. * *p* < 0.05, ** *p* < 0.01, *** *p* < 0.001.

To determine the functional role of T-cell-derived Rcn3 in vivo, we generated T-cell-specific Rcn3 conditional knockout mice using the Lck-Cre/Rcn3^fl/fl^ system (Figure 5A). Efficient deletion of Rcn3 in T cells was confirmed by reduced protein expression in the spleen (Figure 5B).

Following MI, control mice (Rcn3^fl/fl^) exhibited progressive recovery of cardiac function by 21 dpi, whereas Lck-Rcn3 cKO mice showed significantly impaired functional recovery (Figure 5C,D and Table S2). Ventricular wall thickening, including interventricular septum, anterior wall, and posterior wall dimensions, was also attenuated in cKO mice.

Consistent with these functional deficits, expression of fibrosis-associated genes and proteins was significantly increased in Lck-Rcn3 cKO hearts compared with controls (Figure 5E,F). Histological analysis revealed that, although overall fibrosis remained low, the absolute infarct size at 21 dpi was 0.3 ± 0.4% of LV circumference in control mice and 1.3 ± 0.8% in Lck-Rcn3 cKO mice (Figure 5G), indicating a failure to maintain the reparative healing phenotype characteristic of neonatal hearts. Together, these findings indicate that Rcn3 expression in T cells is required to preserve the reparative healing phenotype of the neonatal heart and to prevent a shift toward fibrotic remodeling after ischemic injury.

## 4. Discussion

Cardiac regeneration has been a central topic in cardiovascular research over the past two decades. Although stem cell-based therapies have shown promise in experimental settings, their clinical efficacy has remained inconsistent, with limited and variable improvements in cardiac function [28]. In contrast, accumulating evidence across mammalian species has established the existence of a transient postnatal window during which the heart retains an intrinsic regenerative capacity, characterized by cardiomyocyte proliferation and minimal fibrotic remodeling [2,3,4,5,29,30,31]. However, the mechanisms governing the transition from reparative healing in neonates to fibrotic repair in adults remain incompletely understood.

In the present study, we demonstrate that age-dependent differences in T-cell composition and function critically influence post-infarction cardiac repair. Neonatal hearts exhibited sustained accumulation of CD4+ T cells, CD8+ T cells, and T-reg cells after MI, whereas aging was associated with a marked reduction in CD4+ T cells and T-reg cells and a relative dominance of CD8+ T cells. These findings are consistent with prior reports showing that adaptive immune responses peak approximately seven days after MI in adult hearts [16], but extend this concept by revealing a distinct, neonatal-type reparative immune profile in neonatal myocardium. It should be noted that MI in neonatal mice is characterized by inherently small infarct size and rapid functional recovery, and, consistent with this feature of the model, absolute fibrotic areas remained low even in control animals. This characteristic constrains the resolution with which injury severity and tissue-level outcomes can be interpreted in neonatal MI models.

Previous studies have highlighted divergent roles of T cell subsets in cardiac injury and repair. CD4+ T cells are known to modulate monocyte recruitment, angiogenesis, and extracellular matrix deposition, while excessive IFN-γ production has been implicated in impaired regenerative responses [32,33]. CD8+ T cells have been reported to exert both protective and cytotoxic effects depending on context, including suppression of inflammatory signaling or direct cardiomyocyte injury [34,35]. T-reg cells play a central role in immune resolution, acting through cell–cell interactions and anti-inflammatory cytokine secretion [36,37,38]. T-reg depletion exacerbates post-MI remodeling, whereas adoptive transfer of Tregs enhances cardiomyocyte proliferation, reduces fibrosis, and improves cardiac function in experimental models [39]. Our data support and extend these observations by demonstrating that neonatal T-reg cells display a transcriptional program enriched for processes related not only to immune regulation but also to structural and organelle organization, suggesting a unique reparative phenotype.

Transcriptomic profiling revealed that neonatal T cells, particularly T-reg cells, exhibit broader functional programs than their aged counterparts, including pathways associated with morphogenesis and intracellular organization. Among genes selectively upregulated in neonatal T-reg cells, Rcn3 emerged as a candidate mediator of cardiac repair. Rcn3 expression was enhanced during the acute post-infarction phase in neonatal hearts, implicating it in early reparative processes.

Within the repair–regeneration spectrum, our data position T-cell-derived Rcn3 not as a direct driver of cardiomyocyte replacement, but as a molecular gatekeeper that preserves a neonatal-type reparative state. By restraining fibrotic remodeling and shaping immune–stromal crosstalk, Rcn3 maintains a microenvironment that is permissive for reparative healing. Loss of Rcn3 shifts this balance toward an adult-like repair program dominated by fibrosis and maladaptive remodeling, even in the neonatal heart.

Rcn3 is an ER-resident protein belonging to the CREC family of EF-hand Ca^2+^-binding proteins and has been implicated in protein folding, secretion, and cellular stress responses [25,26]. ER homeostasis is critical for cardiomyocyte survival and adaptation to ischemic stress, and activation of UPR pathways—particularly IRE1/XBP1 and ATF6—has been shown to confer cardioprotection during ischemia and reperfusion [40,41,42,43,44]. In this study, intracellular overexpression of Rcn3 enhanced activation of UPR-associated pathways in T cells under ER stress, whereas treatment with recombinant Rcn3 protein had minimal direct effects on UPR signaling. These data indicate that Rcn3 functions primarily as an intracellular ER-resident protein regulating stress adaptation in T cells. Importantly, the anti-fibrotic effect observed in Figure 4 was not reproduced by recombinant Rcn3 itself, but only by conditioned media from Rcn3-expressing cells, suggesting that Rcn3 indirectly suppresses fibroblast activation by reprogramming the secretory profile of T cells rather than acting as direct extracellular effector. However, we did not directly assess activation of ER stress or UPR pathways in vivo in neonatal or adult hearts after ischemic injury. Thus, the thapsigargin-based experiments should be interpreted as mechanistic in vitro models, and future studies will be required to define the physiological relevance of age-dependent ER stress responses in vivo.

Beyond its role in T cells, Rcn3 exerted a pronounced anti-fibrotic effect on adult cardiac fibroblasts. Conditioned media from Rcn3-expressing T cells suppressed collagen I synthesis in fibroblasts through activation of the PI3K/Akt signaling pathway, consistent with prior observations in human cardiac fibroblasts [27]. These results identify a paracrine mechanism by which T cell-derived Rcn3 indirectly limits fibrotic remodeling of the myocardium. Because ACFs were used in these experiments, this in vitro system does not recapitulate the neonatal post-infarction microenvironment. Rather, it serves as a reductionist model to test whether Rcn3-dependent T cell-derived signals can, in principle, modulate fibroblast activation programs. Age- and injury-specific fibroblast responses, particularly those of neonatal or infarct-derived fibroblasts, remain to be addressed in future studies.

The in vivo relevance of this pathway was confirmed using a T-cell-specific Rcn3 conditional knockout model. Neonatal mice lacking Rcn3 in T cells exhibited impaired recovery of cardiac function after MI, accompanied by increased collagen deposition and expanded fibrotic scar formation. These findings establish T-cell-derived Rcn3 as a critical regulator of neonatal cardiac repair and highlight its role in restraining pathological fibrosis. Importantly, the present study does not directly demonstrate de novo cardiomyocyte generation. We did not assess cardiomyocyte proliferation using markers such as EdU/BrdU, Ki67, or phospho-histone H3, nor did we perform cardiomyocyte lineage-tracing analyses. Therefore, the improved wall thickness and ejection fraction observed in Rcn3-sufficient hearts may reflect attenuated scar expansion, altered inflammatory resolution, or adaptive ventricular remodeling, rather than increased cardiomyocyte replacement. Accordingly, in this study, the term “cardiac repair” is used to denote preservation of the neonatal healing phenotype and suppression of pathological fibrosis, rather than direct evidence of new cardiomyocyte formation. Our data support a model in which T cell-derived Rcn3 promotes immune-mediated repair by limiting fibrotic remodeling and shaping a reparative microenvironment, but do not establish cardiomyocyte regeneration as the primary driver of functional recovery.

Interestingly, Rcn3 contains multiple RxxR motifs that serve as cleavage sites for subtilisin-like proprotein convertases, including PCSK6 [25]. PCSK6 expression is increased in the heart after MI and has been shown to promote collagen synthesis and adverse remodeling when overexpressed in cardiomyocytes [45]. Although the functional interaction between Rcn3 and PCSK6 remains to be fully elucidated, these observations raise the possibility that post-infarction proteolytic processing of Rcn3 may influence its anti-fibrotic activity, warranting further investigation.

Several limitations should be acknowledged. First, we did not include age-matched sham-operated controls. Although cardiac T cells are known to be extremely scarce under steady-state conditions, the absence of baseline data limits our ability to fully distinguish pre-existing age-related differences from injury-induced changes. Future studies incorporating systematic baseline immune profiling across ages will be important to further refine these observations. In addition, immune cell isolation and RNA analyses were performed using whole-heart tissue rather than regionally dissected myocardium. Given the pronounced spatial heterogeneity after myocardial infarction, particularly in neonatal hearts, this approach does not allow precise attribution of Rcn3-dependent effects to the infarct core, border zone, or remote myocardium. Whole-heart measurements may partially reflect changes in immune cell abundance rather than localized molecular responses within regenerating myocardium. Future studies incorporating region-specific analyses will be essential to define the spatial context of Rcn3-mediated repair mechanisms. Second, this study primarily focused on neonatal and aged murine models, and the extent to which these findings translate to adult human cardiac repair remains uncertain. Moreover, although ER stress was experimentally induced in vitro using thapsigargin to interrogate Rcn3-dependent UPR signaling, we did not directly evaluate activation of ER stress pathways in vivo in neonatal or adult hearts after ischemic injury. Third, our in vitro experiments utilized fibroblasts isolated from uninjured hearts. Activated fibroblasts derived from injured myocardium may exhibit distinct ER stress responses and signaling properties. Future studies using injury-derived fibroblasts will be necessary to more precisely define how Rcn3-dependent immune signals modulate ER stress pathways and activation programs in fibroblasts during cardiac repair. Additionally, while our data support a paracrine role for T cell-derived Rcn3 in modulating fibroblast behavior, the precise molecular intermediates downstream of Akt signaling remain to be clarified. Finally, this study does not provide direct evidence of cardiomyocyte regeneration. We did not evaluate cardiomyocyte proliferation using cell cycle markers, nor did we perform lineage-tracing experiments to distinguish newly generated cardiomyocytes from preserved pre-existing cells. Thus, while our data demonstrate immune-mediated repair and attenuation of fibrotic remodeling, they do not establish de novo cardiomyocyte replacement as a mechanism of functional recovery. In addition, interactions between Rcn3 and other immune or stromal cell populations were not explored and may also contribute to the observed reparative phenotype.

## 5. Conclusions

Our findings identify a previously unrecognized role for neonatal T cells—particularly T-reg cells—in cardiac repair after MI. We demonstrate that Rcn3 acts as a key T-cell-derived factor that enhances ER stress adaptation and suppresses fibrotic remodeling through paracrine signaling. These results provide new insights into age-dependent immune regulation of cardiac repair and suggest that targeting T cell-specific pathways such as Rcn3 may represent a novel therapeutic strategy to promote immune-mediated myocardial repair and limit pathological remodeling in the adult heart.

## Data Availability

All data generated in this study, except for raw transcriptome data, are provided in the manuscript and Supplementary Data. Raw transcriptome data are available upon request.

## Supplementary Materials

The following supporting information can be downloaded from: https://www.mdpi.com/article/10.3390/cells15020204/s1, Figure S1. T-cell isolation from post-ischemic hearts using flow cytometry, Figure S2. Bicluster analysis of T cells in post-ischemic hearts, Figure S3. Transcriptome changes after cardiac injury in neonatal and aged hearts using enrichment analyses, Figure S4. Rcn3 gene expression in CD4+ T cells, CD8+ T cells, and CD4+Foxp3+ T-reg cells in the acute and chronic phases of post-injury in neonatal, adult, and aged hearts, Figure S5. ER stress response in Jurkat cells, Figure S6. Detailed components of recombinant lentivirus, Table S1. Forward and reverse primer sequences used in this study, Table S2. Transthoracic echocardiographic measurements.
