# Supplementary material for: Neonatal Regulatory T Cells Mediate Fibrosis and Contribute to Cardiac Repair
Source: Cells. 2026 Jan 22;15(2):204. doi: 10.3390/cells15020204 (PMC12840535; doi:10.3390/cells15020204)
Supplement: Supplementary file 1 [file cells-15-00204-s001.zip › cells-4084821-supplementary.pdf]

Supplementary Data

Neonatal Regulatory T Cells Mediate Fibrosis and Contribute to Cardiac Repair

Tabito Kino, Sadia Mohsin, Yumi Chiba, Michiko Sugiyama, and Tomoaki Ishigami

Supplementary Figures

Figure S1. T cell isolation from post-ischemic hearts using flow cytometry. 2

Figure S2. Bicluster analysis of T cells in post-ischemic hearts. 3

Figure S3. Transcriptome changes after cardiac injury in neonatal and aged hearts using enrichment analyses. 4

Figure S4. Rcn3 gene expression in CD4+ T cells, CD8+ T cells, and CD4+Foxp3+ T-reg cells in the acute and chronic phases of post-injury in neonatal, adult, and aged hearts. 5

Figure S5. ER stress response in Jurkat cells. 6

Figure S6. Detailed components of recombinant lentivirus. 7

Supplementary Tables

Table S1. Forward and reverse primer sequences used in this study. 8

Table S2. Transthoracic echocardiographic measurements. 9

Figure S1. T cell isolation from post-ischemic hearts using flow cytometry. T cell infiltration in **A)** neonatal and **B)** adult and aged hearts.

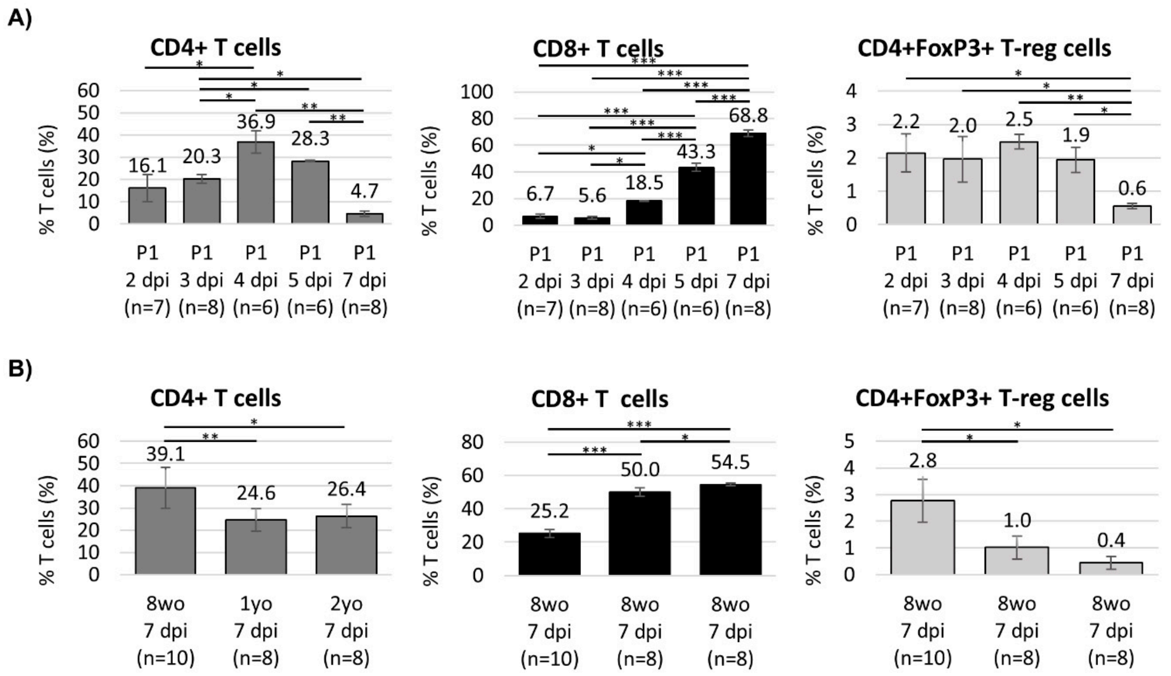

P1, postnatal day 1; 8wo, 8-week-old; 2yo, 2-year-old; dpi, days post-injury. *p<0.05, **p<0.01, and ***p<0.001.

Figure S2. Bicluster analysis of T cells in post-ischemic hearts. **A)** neonatal and **B)** aged hearts.

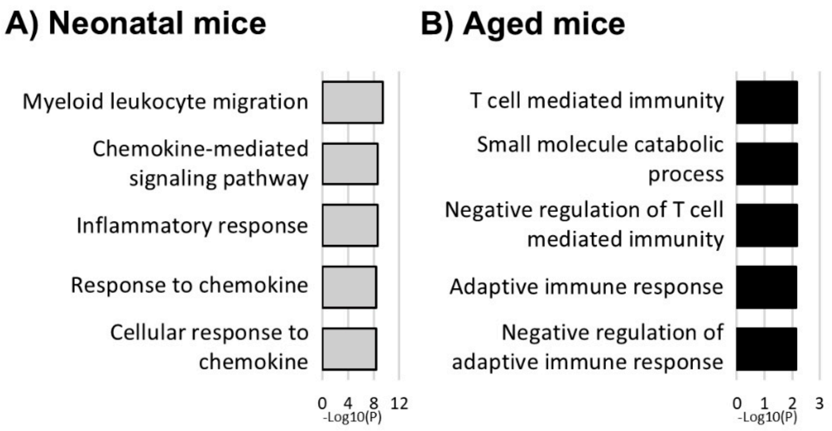

Figure S3. Transcriptome changes after cardiac injury in neonatal and aged hearts using enrichment analyses. **A)** CD4+ T cells, **B)** CD8+ T cells, and **C)** CD4+Foxp3+ T-reg cells.

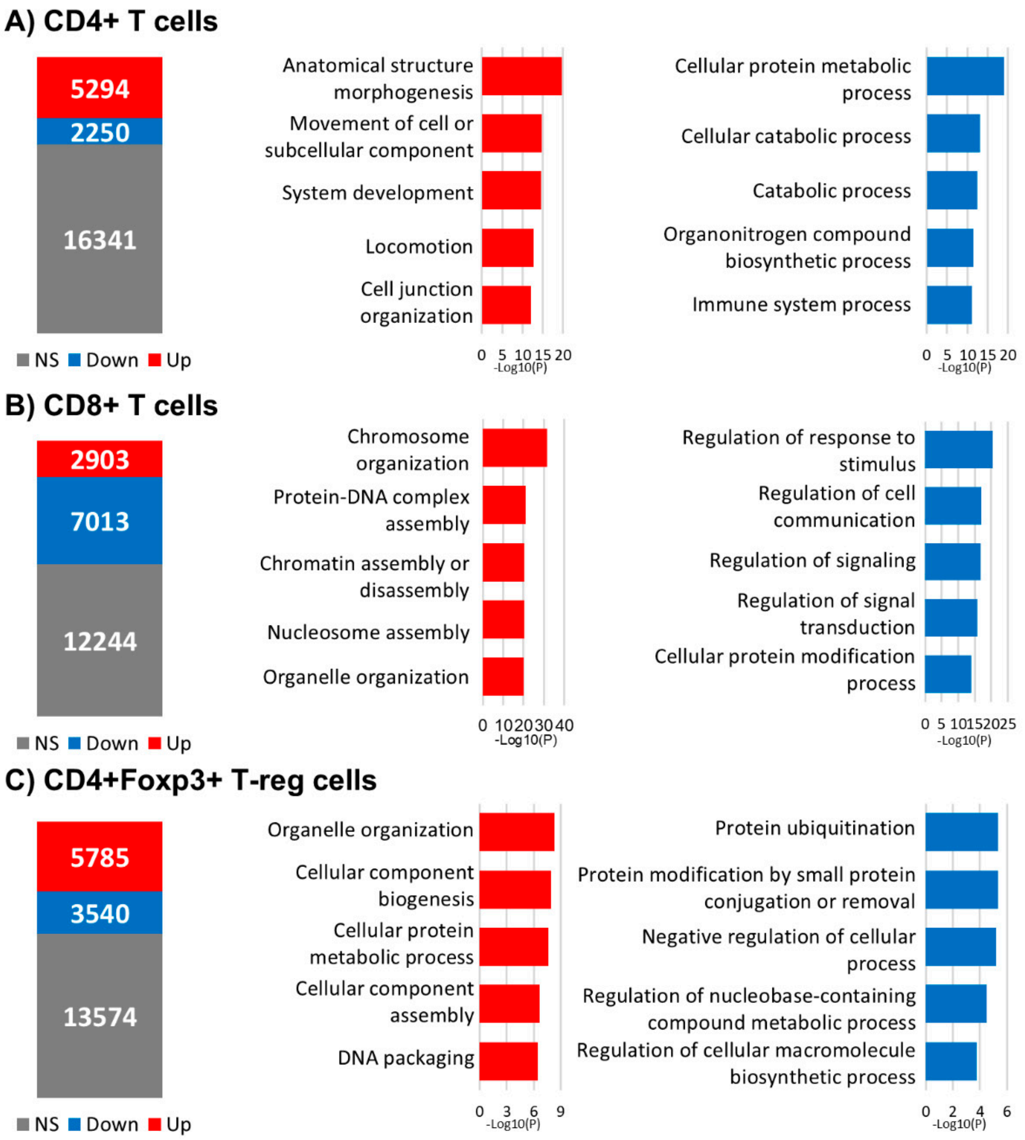

Figure S4. Rcn3 gene expression in CD4+ T cells, CD8+ T cells, and CD4+Foxp3+ T-reg cells in the acute and chronic phases of post-injury in neonatal, adult, and aged hearts.

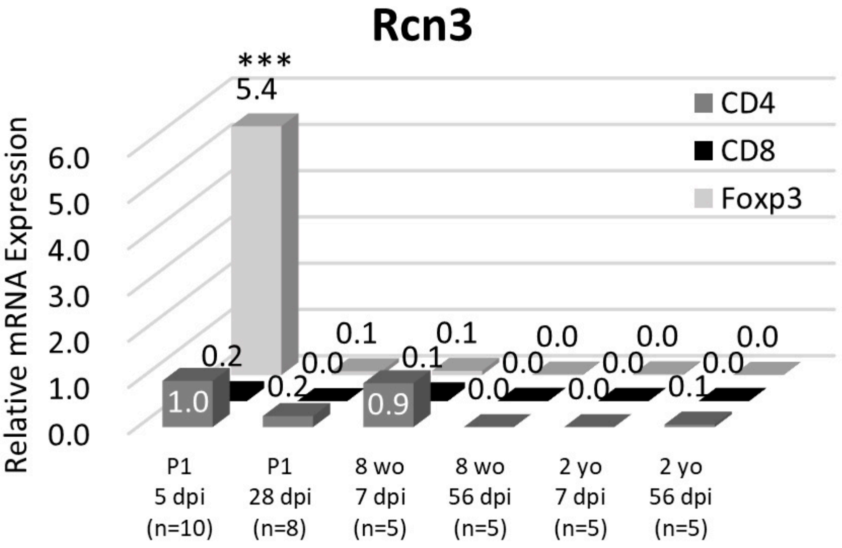

P1, postnatal day 1; 8wo, 8-week-old; 2yo, 2-year-old; dpi, days post-injury. ***p<0.001.

Figure S5. Endoplasmic reticulum **(**ER) stress response in Jurkat cells. **A)** Experimental overview. **B)** ER stress response-associated gene expressions with external Rcn3 treatment.

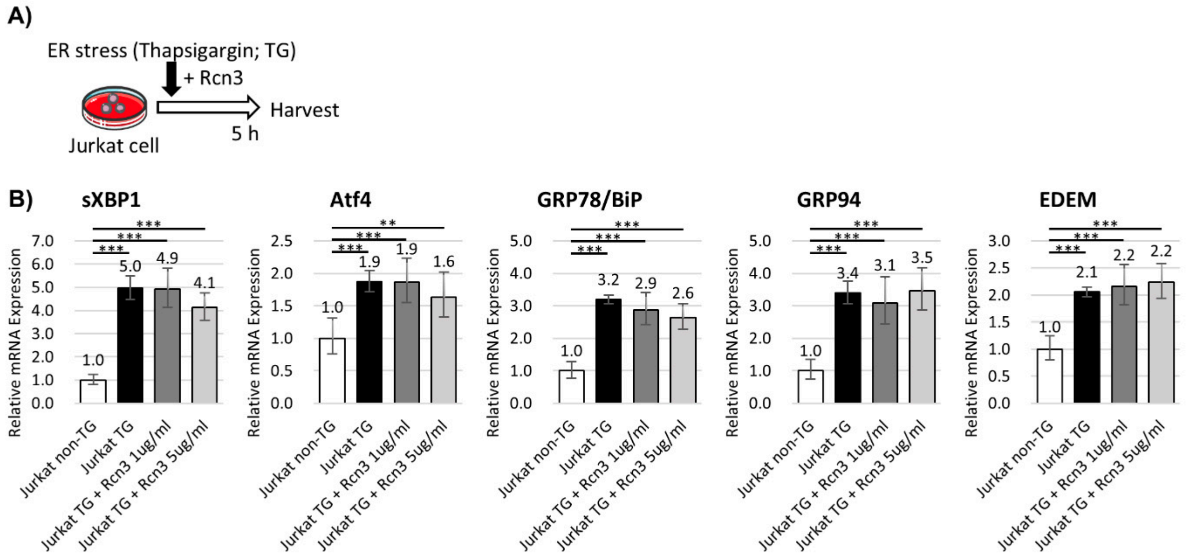

Data represent mean ± SD from n = 3 independent biological replicates. n indicates independent experiments performed on separate days. **p<0.01, and ***p<0.001.

Figure S6. Detailed components of recombinant lentivirus.

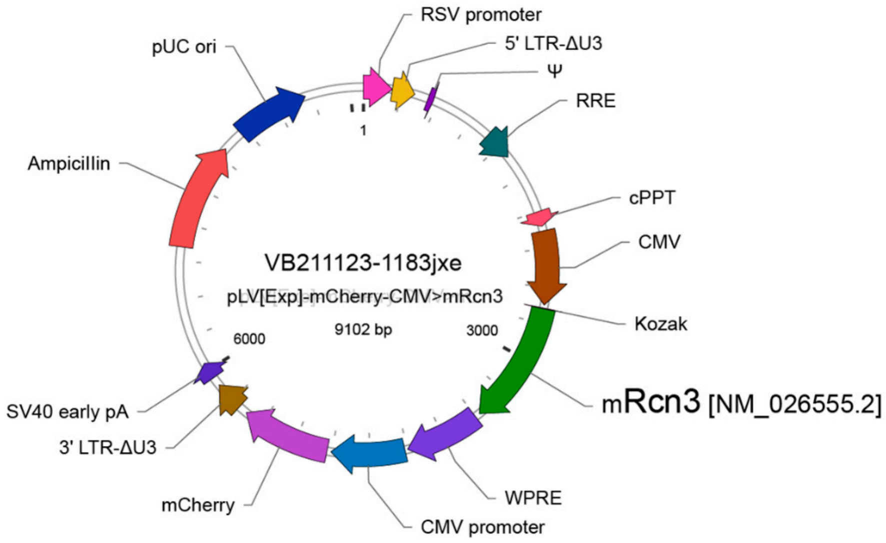

Table S1. Forward and reverse primer sequences used in this study.

Gene	Species	Forward (5'-3')	Reverse (5'-3')
GAPDH	Human	AAGCCTGCCGGTGACTAAC	GTTAAAAGCAGCCCTGGTGAC
GAPDH	Mouse	AGCGAGACCCCACTAACATC	TACGGCCAAATCCGTTCACA
Rcn3	Mouse	CGTAGGGAGCTAACTCAGGC	CCCAGTGCCTCAGCAGTAAG
sXBP1	Human	CTGAGTCCGAATCAGGTGCAG	ATCCATGGGGAGATGTTCTGG
usXBP1	Human	CAGCACTCAGACTACGTGCA	ATCCATGGGGAGATGTTCTGG
totalXBP1	Human	TGGCCGGGTCTGCTGAGTCCG	ATCCATGGGGAGATGTTCTGG
ATF4	Human	GTTCTCCAGCGACAAGGCTA	ATCCTGCTTGCTGTTGTTGG
CHOP	Human	AGAACCAGGAAACGGAAACAGA	TCTCCTTCATGCGCTGCTTT
GRP78/BiP	Human	TGTTCAACCAATTATCAGCAAACTC	TTCTGCTGTATCCTCTTCACCAGT
GRP94	Human	GAAACGGATGCCTGGTGG	GCCCCTTCTTCCTGGGTC
EDEM	Human	CAAGTGTGGGTACGCCACG	AAAGAAGCTCTCCATCCGGTC
PCSK6	Human	GCGCGGGTCATAAAGTTAGC	GCACTAAGGGGATGCTCCTG
Col1a1	Mouse	CCCAGCCGCAAAGAGTCTAC	GGACCCTTAGGCCATTGTGT
Col3a1	Mouse	CCAGTGGCCATAATGGGGAA	ATCTCGACCTGGCTGACCAT
Acta2	Mouse	CAGGCATGGATGGCATCAATCAC	ACTCTAGCTGTGSSGTCSGTGTCG
Tgfb1	Mouse	TCACTGGAGTTGTACGGCAG	TCGAAAGCCCTGTATTCCGT
PD-1	Mouse	CTGGTCATTCACTTGGGCTG	TAGAAGGTGAGGGACCTCCAG
TIM-3	Mouse	GGAGTCTCTGCTGGGTTGAC	TCAGAGCGAATCCTGACTGC
LAG-3	Mouse	GTAGCATCCATCTGCAGGGAC	CAGGGGACGCCACACAAAT

Table S2. Transthoracic echocardiographic measurements.

Parameter	Group	1 dpi	7 dpi	14 dpi	21 dpi	*p*
IVSd	Control	334.8±19.6	452.4±31.1	540.3±29.8	563.1±21.7	**<0.001**
(10^{-3} mm)	cKO	337.2±20.2	412.4±22.4 ***	449.8±60.6 ***	504.8±31.1 ***	
LVIDd	Control	1634.3±149.3	2221.3±206.4	2941.1±145.3	3250.3±265.9	0.743
(10^{-3} mm)	cKO	1611.0±202.1	2218.5±174.4	2880.4±196.2 *	3288.7±288.1	
LVIDs	Control	1369.4±129.1	1720.7±177.0	2211.2±106.9	2202.7±157.1	**<0.001**
(10^{-3} mm)	cKO	1345.2±174.3	1819.8±150.4 ***	2274.0±217.8 **	2600.1±237.4 ***	
LVPWd	Control	339.8±23.1	444.0±22.6	538.4±32.2	569.7±28.3	**<0.001**
(10^{-3} mm)	cKO	337.6±20.7	409.8±21.4 ***	454.6±58.4 ***	500.2±33.6 ***	
AWd	Control	226.2±19.2	340.9±15.0	438.1±15.3	453.1±14.5	**<0.001**
(10^{-3} mm)	cKO	226.8±22.5	278.7±50.2 ***	371.2±46.9 ***	377.9±55.3 ***	
EF	Control	37.1±2.0	48.1±2.9	50.7±1.7	61.6±3.0	**<0.001**
(%)	cKO	37.7±1.3	39.5±5.1 ***	42.6±5.3 ***	45.5±4.7 ***	
FS	Control	16.2±1.0	22.7±1.6	24.8±1.0	32.1±2.2	**<0.001**
(%)	cKO	16.5±0.5	18.0±2.7 ***	20.1±2.9 ***	21.9±2.7 ***	
LV mass	Control	6.8±1.5	16.5±3.4	33.4±3.7	42.3±6.7	**0.008**
(mg)	cKO	6.6±1.1	14.6±2.4	26.2±2.7 ***	35.8±5.7 ***	
EDV	Control	7.7±2.0	16.6±3.9	33.5±4.2	43.0±8.5	0.961
(μL)	cKO	7.5±2.7	16.7±3.3	31.9±5.5	44.3±10.0	
ESV	Control	4.9±1.3	8.7±2.2	15.5±1.9	17.4±2.8	**0.008**
(μL)	cKO	4.7±1.8	10.1±2.1	17.9±4.3 **	24.9±6.0 ***	
SV	Control	1.8±0.3	5.1±0.8	11.8±2.3	17.1±4.4	**0.003**
(μL)	cKO	1.5±0.7	4.1±0.8	7.7±2.5 ***	12.5±2.7 ***	

dpi, days post-injury; cKO, conditional knockout; IVS, interventricular septal thickness; LVID, left ventricular internal diameter; PW, posterior wall thickness; AW, anterior wall thickness; EF, ejection fraction; FS, fractional shortening; EDV, end-diastolic volume; ESV, end-systolic volume; SV, stroke volume. **p<0.01 and ***p<0.001.
